# Supplementary material for: Comparative multiomics analysis of cell physiological state after culture in a basket bioreactor
Source: Sci Rep. 2022 Nov 23;12:20161. doi: 10.1038/s41598-022-24687-4 (PMC9686226; doi:10.1038/s41598-022-24687-4)
Supplement: Supplementary file 1 — Supplementary Information 1. [file 41598_2022_24687_MOESM1_ESM.zip › raw data/Transcriptomic raw data/6.Enrichment/2.KEGG/TE1vsTC1/DOWN/TE1vsTC1.down_KEGGdot.pdf]

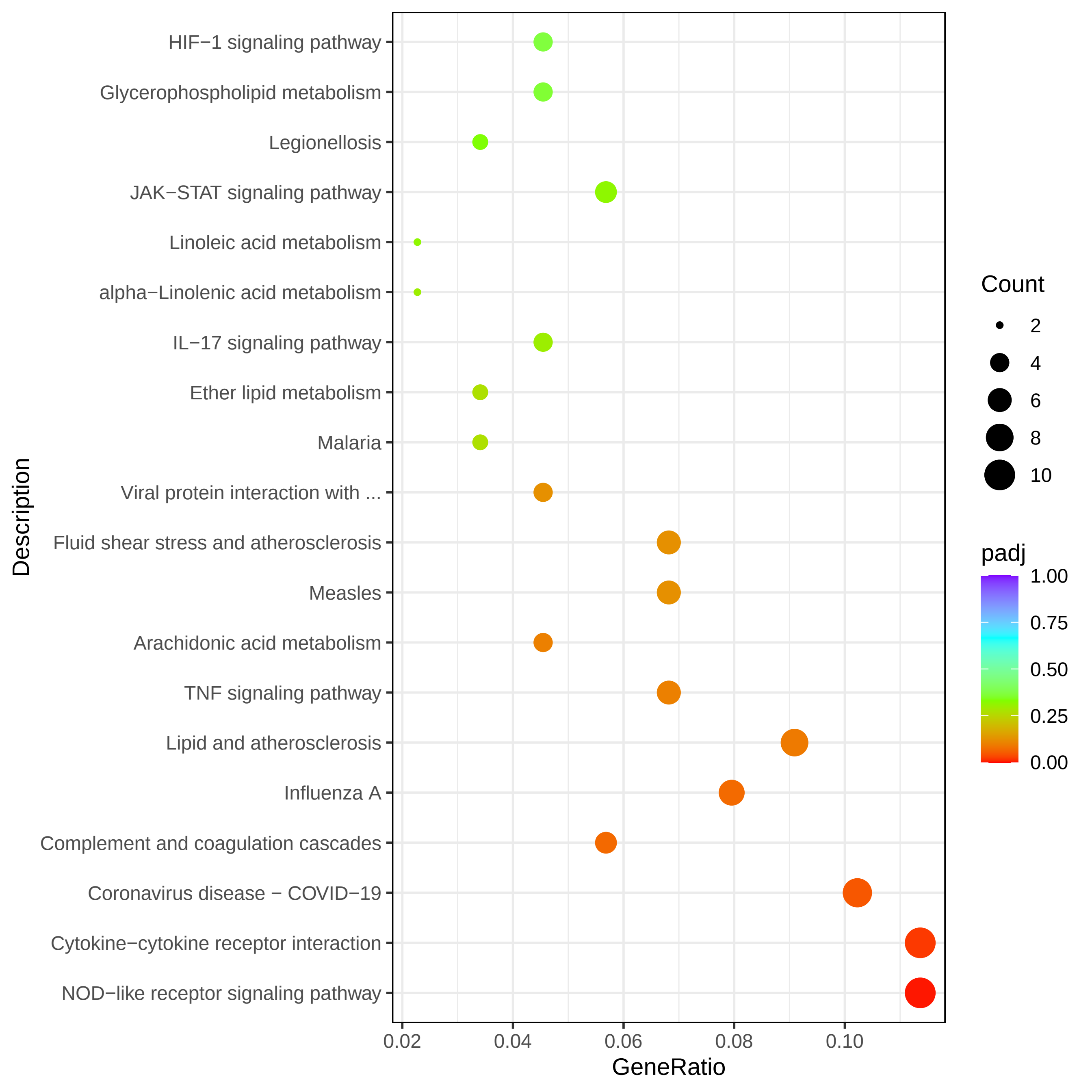

HIF−1 signaling pathway
Glycerophospholipid metabolism
Legionellosis
JAK−STAT signaling pathway
Linoleic acid metabolism
alpha−Linolenic acid metabolism
IL−17 signaling pathway
Ether lipid metabolism
Malaria
Viral protein interaction with ...
Fluid shear stress and atherosclerosis
Measles
Arachidonic acid metabolism
TNF signaling pathway
Lipid and atherosclerosis
Influenza A
Complement and coagulation cascades
Coronavirus disease − COVID−19
Cytokine−cytokine receptor interaction
NOD−like receptor signaling pathway
Description
0.02
0.04
0.06
0.08
0.10
GeneRatio
Count
2
4
6
8
10
padj
1.00
0.75
0.50
0.25
0.00
